# Facial Swelling Mimicking Anaphylaxis: A Case of Superior Vena Cava Syndrome in the Emergency Department

**DOI:** 10.7759/cureus.29678

**Published:** 2022-09-28

**Authors:** Mohamed Elamin Salama, Patrick Ukwade, Abdur Rafeh Khan, Hasan Qayyum

**Affiliations:** 1 Emergency Department, Zayed Military Hospital, Abu Dhabi, ARE; 2 Emergency Department, Sheikh Shakhbout Medical City, Abu Dhabi, ARE; 3 Clinical Imaging, Sheikh Shakhbout Medical City, Abu Dhabi, ARE; 4 Emergency Department, Sheikh Khalifa Medical City, Abu Dhabi, ARE

**Keywords:** catheter-related thrombosis, thrombosis, venous obstruction, superior vena cava syndrome, superior vena cava obstruction, emergency medicine

## Abstract

Superior vena cava (SVC) syndrome represents clinical manifestations from obstruction of the SVC. It is an uncommon medical emergency that is commonly missed. SVC obstruction is usually caused by extraluminal compression by an intrathoracic mass or intraluminal obstruction from a thrombus. The latter is more common in patients with indwelling central venous catheters and pacemaker leads. Here, we present the case of a 53-year-old woman who presented to the emergency department (ED) with clinical features which were initially diagnosed as anaphylaxis and subsequently diagnosed as a case of SVC syndrome. In this case, diagnostic challenges in the ED and the potential role of cognitive bias are highlighted. In addition, we discuss the clinical manifestations and management of SVC syndrome.

## Introduction

Superior vena cava (SVC) syndrome is a medical emergency that can be associated with severe or life-threatening symptoms [1]. In most cases, the SVC is compressed externally by a primary intrathoracic malignant mass, but intraluminal obstruction by a thrombus can also result in similar signs and symptoms of SVC syndrome [2]. Thrombosis-related SVC obstruction can occur in the context of a hypercoagulable state; additionally, clot-related SVC obstruction is common in patients with indwelling central venous catheters and pacemaker leads [2]. Here, we report an unusual case of a patient who presented to the emergency department (ED) with facial swelling and was diagnosed as a case of an allergic reaction and was later found to have SVC syndrome. We aim to highlight that cases of SVC syndrome due to indwelling vascular catheters have increased, accounting for 20-40% of cases of SVC syndrome [3].

## Case presentation

A 53-year-old woman presented to the ED with a three-day history of difficulty breathing, along with face, neck, and tongue swelling and a change in her voice after she used henna, a coloring dye, five days prior. She denied any pain, chills, or pruritus.

Upon further history taking, it was discovered she had been diagnosed with rectal adenocarcinoma with liver metastasis five months prior to her presentation to the ED. Her medical comorbidities included diabetes mellitus and dyslipidemia, both of which were well controlled. She was allergic to penicillin. She had not taken any new medications recently and there was no family history of malignancies. She had recent inpatient admission due to a severe acute respiratory syndrome coronavirus 2 (SARS-CoV-2) infection.

On initial evaluation, her vital signs were as follows: a temperature of 36.8°C, a blood pressure of 140/86 mmHg, a pulse rate of 124 beats/minute, a respiratory rate of 18 breaths/minute, and oxygen saturation of 99% on room air. Clinical examination revealed facial and neck swelling, but no oropharyngeal swelling or urticaria. Henna coloring was noted on both hands. The rest of the systemic examination was within normal limits. Her biochemical and hematological investigations, including D-dimer, were all within normal limits. A 12-lead electrocardiogram (ECG) revealed sinus tachycardia at a rate of 120 beats/minute, and a chest X-ray showed that she had a vascular access port in place, with the tip projected over the SVC, an elevated right hemidiaphragm with right basal atelectasis, and hyperinflation of the left lung. The initial working diagnosis was an anaphylactic reaction to henna, and she was treated with intramuscular (IM) epinephrine, steroids, and antihistamine, with significant improvement in her symptoms. She was discharged with an EpiPen (epinephrine) Auto-Injector, steroids, and antihistamine.

Three days later, the patient re-attended the ED with similar complaints but with the addition of swelling noted in her upper truncal area and hands. She had stopped using henna and the only new medication she had taken was those prescribed at her index visit except the EpiPen (epinephrine) Auto-Injector. On examination, she was found to be tachycardic at a rate of 106 beats/minute with persistent facial and neck swelling extending to the upper trunk. Blood investigations were repeated, which were all within normal limits. A 12-lead ECG again showed sinus tachycardia at a rate of 106 beats/minute. We then suspected SVC syndrome and consulted the on-call radiologist to ascertain which imaging modality would be required to confirm this condition. The radiologist suggested an ultrasound (US) of the internal jugular vein (IJV) and subclavian vein (SCV) as an initial diagnostic test. Given that the patient had a recent computed tomography (CT) of the chest with evidence of SARS-CoV-2 pneumonia one month prior and to limit radiation exposure, he advised that a CT chest with contrast would be the next diagnostic test of choice if US of IJV and SCV are normal and clinical suspicion for SVC syndrome is still high. US of bilateral IJV and SCV revealed patent veins, with no engorgement or thrombus formation. After reassessment, she was discharged from the ED with the impression of an allergic reaction secondary to henna on IM epinephrine and steroids and safety netting.

Four days later, the patient presented to the ED once again with complaints of worsening symptoms, particularly increased difficulty in breathing. Again, she was only noted to be tachycardic at a rate of 112 beats/minute, and other vitals were within normal range. Facial puffiness was still noticeable, with redness and mild neck swelling but no oropharyngeal swelling or urticarial rashes. Fading henna coloration was noted on both hands. Auscultation of her heart and lung sounds was normal. No dilated neck veins were noted. Labs done, including D-dimer, all were within normal limits. An ECG revealed sinus tachycardia.

**Figure 1:**
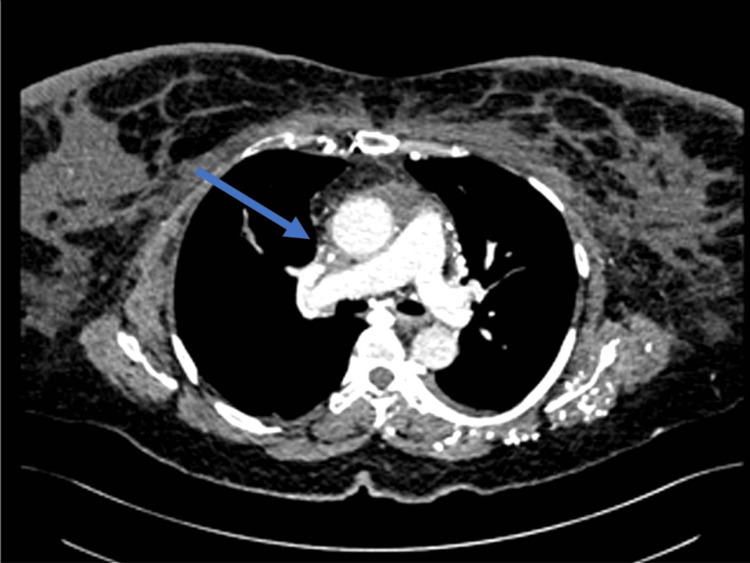
Computed tomography of the chest with contrast showing a filling defect in the superior vena cava at the level of the tracheal bifurcation.

The emergency physician suspected a pulmonary embolism, and a CT pulmonary angiography was performed (Figure 1) which showed thrombosis of the SVC and left brachiocephalic vein.

The patient was admitted to the hospital and commenced on therapeutic low-molecular-weight heparin, which was then switched to a novel oral anticoagulant two days later.

Moreover, stripping off the Port-A-Cath was done by the interventional radiology team as a clot was found when contrast was injected to assess the function of the catheter. The SVC and bilateral brachiocephalic veins showed thrombosis, which was aspirated. Stenosis was found at the junction between the SVC and right atrium and angioplasty with a 14-mm balloon resulted in successful dilatation.

Finally, she was discharged home with outpatient follow-up. On subsequent clinic visits, she reported that her signs and symptoms had completely resolved.

## Discussion

SVC syndrome represents the clinical manifestations of obstruction of the SVC, resulting in decreased venous return from the head, neck, and upper extremities [4]. It was first described by William Hunter in the 18th century in a patient with a syphilitic aortic aneurysm [5]. The obstruction can be due to a neoplastic invasion of the venous wall with associated intravascular thrombosis or by extrinsic compression exerted by a tumor mass against the wall of the SVC. Most cases are caused by malignant mediastinal tumors [4], of which 85% are due to lung cancers, particularly small-cell and squamous cell carcinomas. The non-malignant etiologies include aortic aneurysm, mediastinal fibrosis, thyromegaly, thymoma, Behcet’s syndrome, and infections such as tuberculosis, histoplasmosis, syphilis, and actinomycosis [4]. The clinical presentation of SVC syndrome may include swelling of the face, arms, and neck; dyspnea and cough; and dilated neck veins. Other less common symptoms include headache, syncope, nasal congestion, lingual swelling, hoarseness of voice, dysphagia, epistaxis, and hemoptysis [6]. Syncope is an unusual presentation [4].

SVC syndrome encompasses a wide clinical spectrum, ranging from asymptomatic cases to rare life-threatening emergencies with airway compromise, or increased intracranial pressure, both of which can be fatal [7]. Although direct tumor invasion or external compression remains the most common cause of SVC syndrome, the overall incidence of SVC syndrome associated with indwelling vascular catheters has increased. This is most likely attributed to the increased use of intravascular devices [3]. It is estimated that around 40% of cases of SVC syndrome are secondary to a benign etiology [7]. These patients generally present more acutely in comparison to cases caused by malignant compression due to the rapid rate of thrombosis and lack of venous collateral formation as opposed to the slow progressing obstruction associated with malignancy [8].

SVC syndrome is an uncommon medical emergency and remains a rare diagnosis in the ED [9]. There is a risk of cognitive bias resulting in diagnostic error, as occurred in this case. On the first attendance, perhaps the physicians were prone to anchoring and premature diagnostic closure bias that led to diagnosing the patient with an anaphylactic reaction to henna, which was reinforced by diagnosis momentum in which the patient acted as an intermediate to establish the diagnosis and halted further thinking [10]. The second time, possibly anchoring and fixation bias contributed to the delay in diagnosing SVC syndrome. In the ED, extreme caution should be exercised for any patient who comes with a presumptive prediagnosis [10].

The suspicion of SVC syndrome should be followed by an appropriate diagnostic approach. In our case, we began with US of the IJV and subclavian vein, both of which were patent with no thrombosis. However, it is recommended to additionally include axillary and brachiocephalic veins while using US to evaluate for thrombosis in patients with mild symptoms who have an indwelling device or a malignancy at low risk for causing SVC syndrome [11]. Doppler ultrasound, as well as echocardiography, might reveal an obstructive flow pattern in the proximal subclavian or jugular veins, brachiocephalic veins, or collaterals [12].

Although the SVC itself may be difficult to image with US, the diagnosis can be suspected indirectly at the bedside in the hands of a skilled emergency physician. Operator-dependent point-of-care US is becoming an increasingly available resource [13]. Contrast-enhanced CT venogram is the main diagnostic imaging modality and helps define the degree and cause of SVC obstruction, in addition to the collateral venous supply. It also helps plan the best therapeutic strategy and guide endovascular treatment [14]. Alternatively, contrast-enhanced magnetic resonance imaging may also be used, especially in cases of intolerance to iodine contrast media [7]. The formation of collateral vessels seen either in CT angiography or magnetic resonance angiography can indicate SVC syndrome with a sensitivity and specificity of 96% and 92%, respectively [15]. Venography is generally accepted as the gold standard for visualizing and diagnosing a venous obstruction. This modality can be used along with endovascular intervention for patients with a severe presentation of SVC syndrome [16].

Venous obstruction in the cancer population can result in significant morbidity and mortality [17]. In patients presenting with SVC syndrome, the goal is to treat the underlying cause [8]. Once a patient presents with features suggestive of SVC syndrome, supportive management can be initiated in the ED by elevation of the patient’s head as a simple maneuver with the goal of decreasing venous pressure. Additionally, the use of oxygen and administration of corticosteroids and diuretics initiated in the ED may provide temporary relief pending definitive therapy [18]. Further management is guided by the patient’s underlying SVC syndrome etiology [16]. When the reason for obstruction is a thrombus, the mainstay of treatment is anticoagulation with thrombolytics used as adjuncts [8]. For patients with obstruction due to a malignancy, a multidisciplinary treatment plan depending on the tumor type and stage can help guide the appropriate chemotherapy, radiation therapy, or both [16].

## Conclusions

With reference to our case, when the clinical suspicion for SVC syndrome is high, the patient should undergo time-critical investigations and treatment to avoid further progression of the disease and its complications. Finally, unplanned re-attendances of patients in the ED should prompt senior medical review and should be benchmarked by quality indicators and systemic metrics.
